# Examples of applied public health through the work of the Epidemic Intelligence Service officers at CDC’s National Center for Environmental Health: 2006–2015

**DOI:** 10.1186/s40985-017-0051-x

**Published:** 2017-01-25

**Authors:** Yulia I. Carroll, Fauzia A. Rashid, Henry Falk, Meredith M. Howley

**Affiliations:** 1 Office of the Associate Director for Science, National Center for Environmental Health/Agency for Toxic Substances and Disease Registry, Centers for Disease Control and Prevention (CDC), Chamblee Campus, 4770 Buford Highway, Building 102, MS:F-45, Atlanta, GA 30341 USA; 2 Rollins School of Public Health, Emory University, Atlanta, GA USA; 3 Office of Noncommunicable Disease, Injury and Environmental Health (ONDIEH), Centers for Disease Control and Prevention (CDC), Chamblee Campus, 4770 Buford Highway, Building 106, MS:F-39, Atlanta, GA USA

**Keywords:** EIS, Epidemiology, Environment, Health, Training

## Abstract

The Epidemic Intelligence Service officers (EISOs) at the National Center for Environmental Health (NCEH)/Agency for Toxic Substances and Disease Registry (ATSDR) respond to public health outbreaks, assist public health surveillance, and recommend public health actions. We summarize the breadth of work done by EISOs assigned to NCEH/ATSDR during 2006–2015. We used the Web of Science, Scopus, and PubMed databases to identify articles authored by the EISOs, number and types of epidemiologic assistance field investigations (Epi-Aids), and interviewed NCEH/ATSDR programs with EISO assignees. The largest number of NCEH/ATSDR EISO publications (*n* = 61) and Epi-Aids (*n* = 110) related to toxic chemicals (23 and 37, respectively), followed by natural disasters and those caused by humans (19 and 25, respectively), extreme temperature-related illness (9), and chronic diseases (8). The investigations raised awareness, identified risk factors and public health needs, and introduced better prevention and protection measures for human health. Through field investigations and other technical assistance, NCEH/ATSDR provided leadership and staff scientists to assist in the field, as well as knowledge transfer to local, state, territorial, and international health departments.

## Background

The Epidemic Intelligence Service (EIS) is a 2-year training program in epidemiology and public health [1]. Approximately 80 doctorate-level scientists, medical doctors, health care professionals, and veterinarians are selected to become EIS officers (EISOs) each year. EISOs at the Centers for Disease Control and Prevention (CDC) are a cadre of well-trained epidemiologists who are mobilized on short notice to respond to public health emergencies, solve outbreaks, and recommend protective public health actions nationally and internationally. The EIS program was created to provide a novel epidemiology training—focused more on the applied, competency-based training using primarily on-the-job experience and mentoring and less classroom instruction. Unlike an academic postgraduate training, the EIS training provides an emphasis on epidemiologic training via field investigations, combined with didactic training from CDC headquarters, close collaboration, and additional mentoring and guidance from senior state and local public health officials and senior investigators from other US and international agencies. It also provides experience in the use of communication and management skills while working with the public and media; a broad understanding of how government functions, including in disaster situations; and practical experience in global investigation and engagement in major humanitarian and result-oriented acute responses [2].

The EIS program assigns new officers to various state and local health departments, other CDC field sites, some other federal agencies, and the CDC centers, institutes, and offices, including the National Center for Environmental Health (NCEH) and the Agency for Toxic Substances and Disease Registry (ATSDR). The focus of EIS training at NCEH/ATSDR is for the EISOs to participate and have substantive roles in national and international investigations of disease outbreaks, respond to public health disasters of natural or human origin, and assist public health surveillance for environmental exposures and disease [3].

During the EIS program’s first 60 years (1946–2005), NCEH/ATSDR EISOs conducted 458 national and international environmental investigations [4]. These investigations revealed new or rare epidemic diseases and unexplained syndromes, led to strengthening of government-wide prevention or eradication efforts, and improved responses to natural disasters and those caused by humans [2, 4]. Although NCEH/ATSDR EISOs participate in major surveillance programs and long-term studies, the hallmarks of the program are outbreak responses including epidemiologic assistance investigations (Epi-Aids) [4, 5]. The Epi-Aid investigations are conducted in response to requests from US state and local health departments, other federal agencies, and foreign ministries of health for assistance with urgent public health problems. Examples include requests to assist with emergency responses, investigate infectious and environmental disease outbreaks, and quantify the impact of diseases.

In this report, we summarize published works of the cohort of 2006–2015 NCEH/ATSDR EISOs. We reflected on the nature and unique aspects of EIS training—rapid, sound, applied epidemiology that is focused on generating quick policy-relevant recommendations to reduce morbidity and mortality.

We searched 2006–2015 structured databases for published, peer-reviewed journal articles within Web of Science, Scopus, PubMed, and CDC’s *Morbidity and Mortality Weekly Report* (*MMWR*). We also obtained the number and types of NCEH/ATSDR-led Epi-Aids and consulted NCEH/ATSDR programs on EISOs’ scope of work, publications, and changes that have resulted from such. There were 35 EISOs assigned to NCEH/ATSDR during 2006–2015. Unlike previous summaries of environmental health work performed by EISOs, we limited our search to EISOs at NCEH/ATSDR. We included articles if all of the following criteria were met: (1) an NCEH/ATSDR EISO was listed as an author, (2) the author’s affiliation was listed as either the EIS program or CDC, and (3) the work was done as part of the author’s EIS training. The latest database search was conducted on December 10, 2015.

We also looked for citations of these articles and used the names of the NCEH/ATSDR EISO as Internet search terms with Google Scholar, Google, and Bing search engines. We looked for publications cited with those search engines and any presentations, news, or agency mentions of this work. We read a total of 190 peer-reviewed articles, 61 of which met the inclusion criteria.

## Results

The largest number of NCEH/ATSDR EISO publications during 2006–2015 related to toxic chemicals (23), followed by natural disasters and those caused by humans (19), extreme temperature-related illness (9), and chronic diseases (8). Full list of these publications can be found on the CDC NCEH EIS website [6]. Here, we present a summary and highlight selected studies.

### Natural disasters

Among natural disasters, hurricanes and floods have devastated US coasts for decades. Although the primary focus of the EIS program is on epidemiologic training and field work, the work done by NCEH/ATSDR EISOs has led to:

- Specifying environmental actions, personal protective actions, and disaster-preparedness plans in public health messaging [7, 8].
- Developing health information for Internet sites and other technological alternatives for useful and effective health communication [9].
- Emphasizing the need for pre-disaster risk communication, surveillance, and dissemination of carbon monoxide (CO)-related information as necessary tools for disaster preparedness, response, and prevention. As a result, NCEH added data from the National Poison Data System as another source for the CDC US CO-poisoning surveillance framework, and interventions targeted to high-risk populations were recommended [10]. In 2016, the US Consumer Product Safety Commission (CPSC) published a notice in the Federal Register for a proposed rule to reduce CO-poisoning deaths and injuries associated with portable generators.
- Pre-identification of staff for active mortality surveillance and incorporation of the active mortality surveillance system into operational emergency response guidelines [11].
- EISOs used CDC’s Community Assessment for Public Health Emergency Response (CASPER) toolkit to quickly and objectively quantify the health and assistance needs of disaster-affected populations [12]. Using CASPER, EISOs identified basic needs such as food, shelter, electricity, water, clothing, and protection from vectors of disease, as major needs following natural disaster events [7, 8, 13].

### Toxic chemicals

For decades, EISOs have studied lead exposure and its effects on health [14]. Although childhood lead poisoning in the USA has largely decreased, global lead poisoning appears to be an increasing problem worldwide [15–17]. In 2010, EISOs helped bring international attention to one of the largest episodes of fatal lead poisoning associated with Nigerian gold ore processing [18–20]. In the 12 months before the study, 26% of young children in the surveyed family compounds died and 82% showed symptoms of lead poisoning before death. These blood lead levels represented the highest lead levels ever seen in a CDC lead investigation [21]. These efforts and those of other agencies and countries led to the implementation of new and safer processing techniques that control dust and residual ore wastes, continued blood lead level surveillance, and soil cleanup.

Because of limited expertise and poor regulatory oversight and policy implementation, lead levels can be extraordinarily high within certain communities, as shown by studies in Nigeria. Limited public awareness or regulatory oversight in developing countries leads to repeat instances of strikingly high exposures to a range of toxic chemicals, in addition to lead, and illness in outbreaks that should be preventable (e.g., diethylene glycol-induced renal failure or aflatoxicosis) [4, 14].

The work done by EISOs brought attention to the fact that refugee immigrant children continue to be at risk for increased blood lead levels, whether because they resettle into older housing or consume imported lead-containing products [22] from their countries of origin [23, 24]. More recent studies by NCEH focused on blood lead prevalence among children in Puerto Rico, Pennsylvania, and Indonesia, with participation by EISOs from other CDC centers [25, 26]. The CDC investigations of blood lead prevalence contributed toward community awareness of lead poisoning and resulted in the publication of updated CDC guidelines for lead screening during the domestic medical examination for newly arrived refugees and international adoptions [27]. In addition, CDC provided support to increase education, case management, and environmental follow-up in the children’s countries of origin.

In 2016, NCEH EISOs were instrumental in the CDC public health assistance to the lead-contaminated drinking water crisis in the US city of Flint, Michigan.^1^

In 2006, NCEH EISOs investigated an outbreak of unexplained acute renal failure accompanied by severe neurological dysfunction in Panama [28]. Twelve (57%) of 21 patients died before the contamination of cough syrup with diethylene glycol was discovered to be the cause. This investigation led to the recall of approximately 60,000 bottles of contaminated cough syrup, widespread screening of potentially exposed consumers, and treatment of more than 100 affected patients. This study helped provide guidance on laboratory confirmation and diagnosis, outbreak investigation, awareness of the potential use of mislabeled ingredients in pharmacies, and prevention.

In 2012, NCEH EISOs were one of the first in the USA to identify laundry detergent pods eaten by children to be the cause of adverse gastrointestinal, respiratory, and mental health outcomes [29]. This and research by others led in 2016 laundry detergent company ad campaigns to emphasize safe use of the packets and packaging made more difficult for children to open. In 2013, NCEH EISO together with FDA staff identified 92 cases of poisoning as a result of a new ingredient in a dietary supplement OxyELITE Pro® (OEP) [30]. As a result of this investigation, the manufacturer recalled its products in the USA and abroad and national retailers removed it from their shelves. This investigation was the first to recognize OEP as a potential cause of liver injury. It is one of the largest reported outbreaks of liver disease associated with a dietary supplement to date. NCEH EISOs were also among the first to raise awareness about poisoning associated with the use of electronic cigarettes, more than half of these cases involved children under the age of five. They reported a greater than 1400% increase in the annual e-cigarette calls—from 238 in 2011 to 3692 in 2014 [31, 32]. These are examples of how modification of an established product can lead to unexpected problems.

### Extreme temperature

Investigations of climate change-related illness such as extreme cold and heat have increased. For example, EISOs’ reports of sport-related heat illness were followed by the first reporting of national estimates of heat illness among all sports and recreation participants, an estimated 5946 emergency department annual visits [33]. Through this, the NCEH Health Studies Branch raised prevention awareness among athletes, coaches, athletic trainers, and parents and guardians. The branch also developed an Internet-based training course and educational materials on ways to prevent heat-related illness [34].

### Field investigations (Epi-Aids)

Many of the publications included in this review were based on results from Epi-Aids (Table 1). The number of all CDC-wide Epi-Aids has dropped over the years, from 1018 during 1976–1985, 907 during 1986–1995, to 846 during 1996–2005 [35]. Those led by NCEH/ATSDR followed a similar trend: 126 (12.4% of all Epi-Aids) during 1976–1985, 101 (11.1%) during 1986–1995, and 69 (8.0%) during 1996–2005.

**Table 1:** Number of Epi-Aids performed by NCEH EISOs, by subject area and year, 2001–2015

Subject area	2001–2004	2005–2009	2010–2015	Total
Toxic chemicals	15	12	25	52
Indoor air quality and outdoor air toxics	2		2	4
New, rare, or unexplained disease/syndrome	12	8	2	22
Natural disasters	9	12	7	28
Terrorism and unintentional human-made disasters	5	1	5	11
Substance use and abuse		2	4	6
Violence and injuries	1		1	2
Infectious disease	17	11	12	40
Miscellaneous	2	4	4	10
Total	63	50	60	175

Data provided from personal communication with the CDC EIS Program

During 2005–2014, other, relatively newer, CDC centers took on many subject matter expertise areas in Epi-Aids that previously would have been led by NCEH/ATSDR, such as occupational health, unintentional injuries and violence, global emergency response, and refugee health. Even so, the number of Epi-Aids led by NCEH/ATSDR during the period increased to 110. This might reflect the increased number of Epi-Aids to investigate toxic chemical spills. Toxic chemical Epi-Aids are led by the HSB CASPER and the ATSDR Assessment of Chemical Exposures Program, a component of the National Toxic Substance Incidents Program [36]. Since 2010, the program increasingly has been using EISOs to lead the investigations. There has also been an increase in investigations related to emerging new toxins such as those in dietary supplements [37], drug overdose deaths [38], and electronic cigarettes [32]. Although only seven Epi-Aids for toxic chemicals were conducted during 1996–2005 [4], 37 were conducted during 2005–2015 (Table 1). In addition, the response to natural disasters such as hurricanes have required deployment of great numbers of EIS officers, such as in 2005, 98 EIS officers were sent to evacuation centers and areas affected by Hurricane Katrina. In 2012, EISOs assisted the American Red Cross with disease surveillance in shelters after Hurricane Sandy. There were 400 US Public Health Service commissioned corps officers deployed. EISOs’ studies of health related to climate change, such as extreme heat and cold, air quality and asthma prevention, and health prevention during natural disasters, have increased during the years.

Many investigations are still not published. These include investigations into synthetic marijuana, steroid-laced vitamins, arsenic in private well water, private well water contamination, and chemical spills and pesticide exposures, in the USA and internationally. Other investigations were conducted by non-NCEH/ATSDR EISOs supervised by NCEH/ATSDR. This occurs when NCEH/ATSDR EISOs are already involved in other field investigations. In these situations, publications of environmental health Epi-Aids were not included in our search of publications. Most NCEH Epi-Aids are led by the NCEH Health Studies Branch and are reported on their website [5]. The reports from the Epi-Aid investigations are usually prepared within a month and often are posted on the websites of the state or local health departments that requested the Epi-Aid.

## Discussion

EISOs’ work during 2006–2015 addressed a broad range of important environmental health issues and often built on work done by previous EISOs. Each investigation provided opportunities to refine or add new, detailed information to update standard practices for prevention and response. Their investigations also helped shape the public health focus at NCEH, such as the recent new emphasis on electronic cigarettes and drug overdoses.

Exposures to natural disasters and toxic chemicals, such as lead, continued to be the leading driver of investigations by the NCEH/ATSDR EISO (as identified to be during the first 50 years of the program (4)). There were new topics that emerged in the last 10 years—climate-related investigations into health effects of extreme cold and hot weather and natural disasters, well-known chemicals in new market products such as laundry pods and electronic cigarettes, and an increase in dietary supplements and drug overdose deaths. This report also highlights increasing engagement in disaster epidemiology, global investigations, and inclusion in CDC investigations beyond those led by NCEH/ATSDR. During 2014–2015, for example, all EISOs were engaged either domestically or in West Africa as part of CDC’s Ebola virus response. At the same time, NCEH/ATSDR EISOs have been actively engaged in major environmental investigations.

Although the main focus of the epidemiologic training for NCEH/ATSDR EISOs is environmental health, the opportunity to assist in field investigations led by other CDC centers can lead to much broader experience and training in epidemiologic methods. For example, after the 2010 earthquake in Haiti, NCEH EISOs worked with other CDC centers to develop the National Sentinel Site Surveillance System [39]. The surveillance system has proven useful in allocating resources and identifying effective public health interventions. The system also highlighted the need for additional surveillance education for health care providers and need for increased capacity to perform laboratory diagnostic testing and Internet-based reporting. This investigation, like many of the others described in this review, pointed to the increased use of the Internet for dissemination of disaster-related news. Studies such as these have led CDC and other federal agencies to include updated disaster-related information on their websites and to use social media such as Twitter, Facebook, and smartphone applications to distribute public health messages. They have also helped with the acceptance and development of electronic health records and Internet-based surveillance.

Through field investigations and other technical assistance in which EISOs participate, NCEH/ATSDR also provides knowledge transfer to local, state, territorial, and international health departments. For example, during the Nigeria lead investigations, the CDC team provided extensive training in epidemiologic and sampling techniques to local health workers and staff at national ministries of health. This helped build public health capacity so the work can continue for years after the CDC team leaves.

Being an EIS officer at NCEH/ATSDR also means acquiring practical experience in specific subject areas in environmental health, such as air pollution, water pollution, water- and food-borne disease outbreaks (such as on cruise ships), radiation studies, toxicology, risk assessment, and management of solid and hazardous wastes. NCEH/ATSDR EIS officers represent on average 3% of all EIS officers. Yet, a recent study found that 8% of all government public health workers are environmental health workers and 2% are epidemiologists [40]. This shows the high need for training in environmental health in the current public health workforce.

Studies have pointed out that some of the most important attributes for career success among epidemiology trainees in the USA are being trained in a high-quality program, passion to improve population health (and thus opportunities to improve such), and having a supportive mentor [41]. The top competencies were skills working in multidisciplinary teams and in using modern information technologies. All of these are the cornerstones of the EIS program: hands-on learning by responding to outbreaks/disasters and recommending/implementing population-level changes, close mentorship, and providing support for national- and state-level decision-making. Upon completion of the program, nearly 90% of EISO alumni continue in public health careers at the local, state, federal, or international level, and thus, the EIS program is fundamental in building national and international public health capacity [2]. Because of the heavy emphasis on practical experience while serving the local hosting program has shown to be most successful in acquiring skills (and also being of greatest service to the hosting program), many other training programs have been modeled after the EIS—state EIS programs (such as the Florida EIS program [42]), over 50 international field epidemiology training programs [43], and the CDC/Council of State and Territorial Epidemiologists (CSTE) Applied Epidemiology Fellowship Program [44].

There are still areas for improvement. Although EIS officers provide ongoing information on the Epi-Aids they partake in, it takes over 2 years for manuscripts to be published after an Epi-Aid occurs [45]. Thus, many of these investigations have not resulted in publications or in timely ones. In addition, publication in scientific journals is not a direct measure of the actual public health impact. Many of these publications reflect the ongoing work of public health professionals at federal, state, and local levels. EISOs at NCEH/ATSDR are hosted within a particular NCEH program and gain mainly experience in a specific environmental health area, e.g., air pollution and respiratory disease or emergency response. Some EISOs prefer to gain this type of specific subject matter expertise and continue to work in the area, while others participate in investigations led by other NCEH/ATSDR programs or other CDC centers (such as the international Ebola and Zika responses) and gain much broader public health experience. Ultimately, this enriches both the EISO experience as well as serves the public health in general having both specialized and versatile workforce.

## Conclusions

Public health prevention and disease control depend on how well trained, adequately organized, and supported the public health workforce is. The work of EISOs at NCEH/ATSDR during 2006–2015 covered a broad range of diseases and risk factors, spanning beyond pure environmental risk factors. The epidemiologic training and field investigations, with mentoring by supervisors, provide significant opportunities for hands-on experience and professional growth. The investigations also had very practical benefits, as they raised awareness, discovered risk factors, identified public health needs, and introduced better prevention and protection measures for human health.

## Abbreviations

ATSDR: Agency for Toxic Substances and Disease Registry
CASPER: Community Assessment for Public Health Emergency Response
CDC: Centers for Disease Control and Prevention
CO: Carbon monoxide
EIS: Epidemic Intelligence Service
EISO: Epidemic Intelligence Service officer
MMWR: Morbidity and Mortality Weekly Report
NCEH: National Center for Environmental Health
US: United States

## Footnotes

1 2016—Public Health Emergency Response (CASPER) after the Flint water crisis: May 17−19, 2016. https://www.cdc.gov/nceh/hsb/disaster/casper/states/mi.htm.
